# An improved roosters algorithm for constrained 3D UAV path planning in urban environments

**DOI:** 10.1038/s41598-025-24752-8

**Published:** 2025-11-20

**Authors:** Mashar Cenk Gençal, Barış Ata, Mehmet Kurucan, Emre Kılınç

**Affiliations:** 1 Management Information Systems, Osmaniye Korkut Ata University, 80000 Osmaniye, Turkey; 2 Computer Engineering Department, Cukurova University, 01250 Adana, Turkey; 3 Computer Engineering Department, Adana Alparslan Türkeş Science and Technology University, 01250 Adana, Turkey; 4 Computer Programming Department, Agri İbrahim Cecen University, 04100 Ağrı, Turkey

**Keywords:** Metaheuristic algorithms, Multi-objective optimization, Path planning, Unmanned aerial vehicles, Engineering, Mathematics and computing

## Abstract

Urban environments impose complex challenges for the navigation of unmanned aerial vehicles (UAVs), including dense obstacles, no-fly zones, energy constraints, and regulatory restrictions. Addressing these challenges requires efficient and robust optimization techniques. This study introduces the Improved Roosters Algorithm (IRA), a novel metaheuristic inspired by the natural dominance behavior of roosters, tailored for constrained 3D UAV path planning in urban scenarios. Unlike existing metaheuristics, IRA introduces a spiral dancing operator, adaptive constraint handling, and a hierarchical population structure. These innovations directly target the lack of adaptive mechanisms in constraint-rich urban environments, enabling more reliable and realistic UAV path planning. The performance of IRA is benchmarked against Particle Swarm Optimization (PSO), Standard Genetic Algorithm (SGA), Differential Evolution (DE), Grey Wolf Optimizer (GWO) and the original Roosters Algorithm (RA) across three increasingly complex simulation scenarios. Experimental results demonstrate that IRA consistently outperforms the baseline methods in terms of feasibility and optimality, validating its potential as a competitive tool for UAV mission planning in realistic urban environments.

## Introduction

The utilization of unmanned aerial vehicles (UAVs) has expanded significantly in recent years across both military and civilian sectors, positioning autonomous and safe flight as a critical research priority ^1,2^. Beyond traditional defense applications, UAVs are increasingly deployed in diverse missions such as logistics and delivery services^3^, search and rescue operations, environmental monitoring^1^, and infrastructure inspection^4^. The success of these missions depends heavily on the ability of UAVs to generate optimal flight trajectories while ensuring collision-free navigation in highly dynamic and uncertain environments. Among these, urban airspaces present particularly demanding operational challenges due to dense building clusters, restricted air corridors, and heterogeneous obstacles ^3^.

Given these complexities, UAV path planning has evolved as a multi-constrained optimization problem that is essential for ensuring safe and efficient autonomous flight. Optimal trajectory determination requires more than identifying the shortest distance between an initial and a target location; it must also satisfy dynamic flight constraints, minimize energy consumption, preserve path smoothness, comply with altitude regulations, and incorporate mission-specific requirements. Ineffective planning may result in collisions with obstacles, inability to fulfill operational objectives, or inefficient utilization of limited onboard resources.

**Fig. 1:**
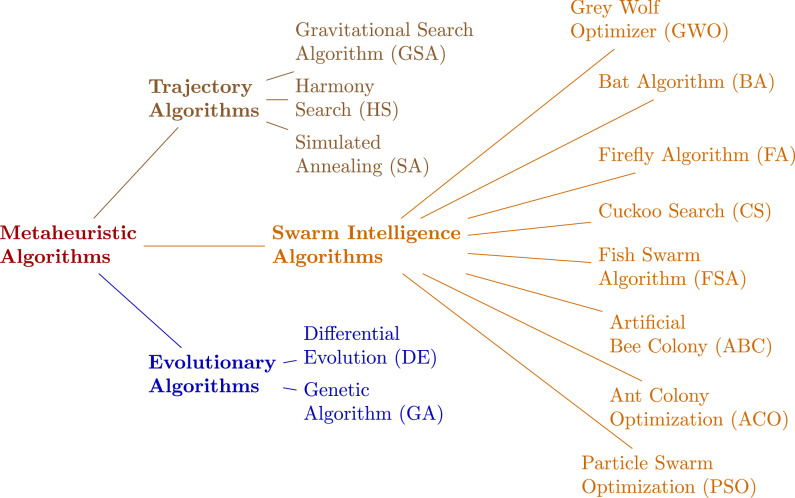
The classification of metaheuristic algorithms used in studies for path planning of UAVs.

Numerous approaches have been proposed to address the challenges of UAV path planning, including classical algorithms such as the Rapidly-exploring Random Tree (RRT)^5^, Artificial Potential Field (APF)^6^, and Voronoi-based methods^7^. While these methods are relatively straightforward to implement, they often fail to ensure reliable performance in complex and dynamic environments and may struggle to generate truly optimal paths. To overcome these limitations, metaheuristic algorithms–robust optimization methods inspired by natural and social phenomena–have emerged as powerful alternatives for UAV path planning, offering flexibility, strong global search capability, and robustness in complex and dynamic environments, as illustrated in Fig. 1.

Representative techniques such as Genetic Algorithms (GA)^8^, Particle Swarm Optimization (PSO)^9^, Differential Evolution (DE)^10^, Ant Colony Optimization (ACO)^11^, and Grey Wolf Optimizer (GWO)^12^ have been extensively applied in UAV path planning, either in their original forms or as part of hybrid frameworks.

Puente-Castro et al. introduced a GA-based path planning strategy that ensures complete coverage of areas with static obstacles while minimizing flight time, regardless of map size or the number of UAVs^13^. Similarly, Gutierrez-Martinez et al. developed a GA-based optimal path planning scheme in three-dimensional environments, emphasizing real-time performance and dynamic obstacle avoidance^14^. On the other hand, Freitas et al. combined Non-Uniform Rational B-spline (NURBS) with the DE algorithm and incorporated the Velocity Obstacle framework to achieve safe online navigation^15^.

Furthermore, recent research has increasingly focused on applying these methods in three-dimensional environments. Fang and Hui proposed an improved nature-inspired DE algorithm tailored to UAV path planning, demonstrating high robustness and adaptability with respect to path length, fitness evaluation, and statistical consistency^16^. The search for enhanced performance also drives the integration of competitive mechanisms into established frameworks; for instance, Zhong et al. introduce a competitive selection process to boost exploration and improve the prioritization of potential solutions^17^. Recent SOTA methods demonstrate the growing need for specialized algorithms in high-dimensional and complex settings. For instance, Zhang et al. applied the Bald Eagle Search (BES) algorithm successfully to generic three-dimensional path planning problems^18^. Building on this, the Enhanced Adaptive Strategy-based Bald Eagle Search (EAB-BES) algorithm was specifically proposed for efficient path planning in high-density urban environments, directly addressing the complexities of cluttered and constrained airspace^19^. Hybrid methods have also drawn significant attention. Ali et al. designed a framework that integrates DE with Maximum-Minimum Ant Colony Optimization (MMACO) algorithms^20^. This framework aims to address the shortcomings of classical ACO by optimizing coordination among multiple UAVs. Along the same line, Haghighi presented a hybrid algorithm that combines PSO and the GA for the Open Traveling Salesman Problem (OTSP) for multi-UAV operations^21^.

Swarm intelligence (SI) algorithms, a prominent and widely adopted class of metaheuristic optimization methods, have also been extensively employed for effective navigation planning in UAVs. To address the weak convergence capability of ACO, one of the most widely applied SI algorithms, Mai et al. proposed a dual-strategy ACO (DSACO) algorithm^22^. Similarly, Bui et al. introduced a cooperative inspection path planning (C-IPP) framework based on ACO that leverages multiple UAVs to solve inspection problems by formulating the task as an extended traveling salesman problem (TSP)^2^. In addition, Ahmed et al. presented an adaptive ACO-based algorithm that enables UAVs to determine optimal paths in dynamic environments^23^.

GWO, another well-known SI algorithm, has gained popularity in recent years, particularly in hybrid variants. Yang et al. proposed the Hybrid Crossover Game and Grey Wolf Optimization (HCGO) algorithm, which combines crossover game optimization (CGO) with GWO, significantly enhancing exploration and exploitation capabilities while reducing the tendency to become trapped in local optima^1^. Addressing the inherent limitations of GWO, including premature convergence, vulnerability to local minima, and limited adaptability in dynamic environments, Nayeem et al. developed a Q-learning-based hybrid algorithm known as Q-learning-based GWO (QGWO)^24^. Likewise, Zhou et al. introduced the Novel Improved GWO (NI-GWO), which enhances convergence speed, robustness, and resistance to local optima in UAV path planning^25^.

PSO, inspired by the social behavior of swarming organisms, is also frequently applied in UAV path planning due to its flexibility, fast convergence, and ability to solve complex optimization problems. Cheng et al. proposed an improved PSO variant, called LGPSO, to handle the challenges of UAV path planning in dense urban environments with dynamic obstacles^3^. Duong et al. presented a navigation-variable-based multi-objective PSO (NMOPSO) algorithm tailored to UAV path planning with kinematic constraints^4^. In another direction, Rosas-Carrillo et al. focused on UAV exploration missions in real-world environments and introduced an adaptive inertia weight strategy, known as KPSO, to enhance PSO’s performance^26^. Extending beyond standard PSO variants, significant effort has been invested in developing hierarchical and hybrid structures to enhance search diversity and convergence. A novel promotive particle swarm optimizer with double hierarchical structures was proposed by Zhang et al. ^27^, drawing inspiration from social and biological competition mechanisms to ensure fair particle competition. In the context of dynamic environments, the integration of swarm intelligence with robust mechanisms for responding to environmental changes is crucial for applications like UAV-assisted Mobile Edge Computing (MEC). For instance, a swarm intelligence-based optimization framework was proposed for UAV-assisted wireless-powered MEC systems aimed at maximizing the computation rate of user devices in dynamic settings. Chen et al. ^28^ enhance population diversity to overcome premature convergence by integrating PSO and harmony search (HS), resulting in the algorithms DOPSO and DOHS, respectively. Simulation results indicated that this proposed dynamic framework significantly outperforms other dynamic optimization algorithms. Furthermore, recent research has focused on introducing entirely novel bio-inspired strategies to overcome the inherent limitations of established MAs. Xu et al. worked on algorithms such as the Crested Ibis Algorithm (CIA) proposes new mechanisms for dynamic exploration and exploitation balance through modeling natural predatory behaviors^29^. Following this trend, Zhong et al. proposed advanced extensions like the Dual Gene Targeting Crested Ibis Algorithm (DGTCIA) integrate tailored operators specifically to address issues of premature convergence and stagnation in complex optimization tasks^30^. Beyond single-objective methods, significant effort has been directed toward Multi-Objective Optimization (MOO) to effectively manage the inherent trade-offs in UAV path planning (e.g., path length vs. safety). Specifically, multi-objective versions of novel metaheuristics, such as the Multi-Objective Bald Eagle Search (MOBES) algorithm, have been developed by Zhang et al., employing Pareto-optimal solution archives to handle these complex design problems^31^.

Unlike prior studies that primarily focus on combining well-established metaheuristics into hybrid frameworks or tuning standard operators for specific scenarios, this work introduces a fundamentally new behavioral operator into the optimization process. The Roosters Algorithm (RA) was chosen as the baseline optimizer for several reasons. First, RA belongs to the class of population-based swarm intelligence algorithms, offering a simple structure with few control parameters, which makes it particularly suitable for systematic modification and extension. Second, despite its structural simplicity, RA has demonstrated competitive performance in continuous optimization problems, providing a solid yet flexible foundation for developing improved variants. Third, in our previous work^32^, RA served as the starting point for designing the Bipolar Improved Roosters Algorithm (BIRA); during the peer-review process, ablation experiments were requested to evaluate the effect of bipolarity and improvement mechanisms separately. This led to the formulation of the Improved Roosters Algorithm (IRA) as an intermediate variant, which exhibited promising results. In contrast to the earlier study where IRA was tested only for comparison, the present work formalizes IRA as a standalone algorithm and applies it to the UAV path planning problem, thereby highlighting its potential in constraint-rich optimization tasks.

The proposed Improved Roosters Algorithm (IRA) extends the original RA through three key innovations: a spiral dancing mechanism that enhances exploration diversity, an adaptive constraint-handling strategy that balances conflicting mission objectives, and a hierarchical population structure that preserves elite solutions while mitigating premature convergence. These mechanisms directly address the lack of adaptive strategies for managing constraint-rich and dynamically complex urban airspaces. The main contributions of this study are as follows:(i) Formalization of the IRA as a standalone metaheuristic with adaptive and hierarchical features. (ii) Comprehensive evaluation of IRA against baseline algorithms across varying urban densities under realistic operational constraints. (iii) Demonstration that IRA consistently achieves the lowest median cost while maintaining robustness across trials.(iv) Validation of IRA’s applicability to practical UAV missions such as urban logistics, emergency response, and infrastructure inspection, where safe and regulation-compliant path planning is critical.

The remainder of the paper is organized as follows: Section details the problem formulation, cost function design, and the proposed IRA algorithm. Section presents the experimental setup, performance evaluation metrics, and comparative results. Section concludes the paper and outlines directions for future research.

## Method

This section presents the proposed approach to solve the urban UAV path planning problem under multiple operational constraints. First, we define the path planning problem in a 3D environment and introduce a multi-objective cost function that incorporates various performance and safety metrics. Then, we describe the IRA, a novel bio-inspired metaheuristic that enhances both exploration and exploitation phases through a spiral-based mating mechanism.

### Problem statement and multi-objective cost function

Urban airspace imposes strict geometric, energetic, and regulatory constraints on UAV operations^33^. We consider a 3D path planning problem for an autonomous UAV tasked with navigating from a start point $S=(x_s, y_s, z_s)$ to a goal point $G=(x_g, y_g, z_g)$ in a cluttered urban environment. This environment contains obstacles such as buildings, modeled as closed volumes collected in the set $\mathscr {O}$, as well as no-fly zones defined by aviation authorities and represented by the restricted set $\mathscr {N}$. The feasible flight region $\mathscr {F}$ is defined as the complement of these forbidden volumes:

1 $$\begin{aligned} \mathscr {F} = \mathbb {R}^3 \setminus (\mathscr {O} \cup \mathscr {N}), \end{aligned}$$

ensuring that the UAV remains entirely within $\mathscr {F}$ for a safe trajectory. The path is modeled as a continuous curve $\textbf{r}(s): [0,1] \rightarrow \mathscr {F}$, which is discretized in practice into a sequence of waypoints $\{P_0 = S,\, P_1, \dots , P_N = G\}$, where each waypoint $P_k \in \mathscr {F} \subset \mathbb {R}^3$. This discretization results in a piecewise linear trajectory and allows for efficient checking of obstacle clearance between consecutive waypoints^34^.

Path planning is formulated as a constrained optimization problem. The objective is to minimize a composite cost *J* while ensuring that all waypoints and connecting segments lie entirely within the feasible space $\mathscr {F}$. We adopt a weighted-sum multi-objective cost function that integrates several performance criteria into a unified objective, a widely used strategy in recent UAV path planning research^35,36^:

2 $$\begin{aligned} J = w_L F_L + w_S F_S + w_H F_H + w_E F_E + w_W F_W + w_O F_O + F_Z, \end{aligned}$$

where each $F_{*}$ denotes a cost component corresponding to a specific operational objective, and $w_{*} \ge 0$ is its associated weight. By adjusting these weights, different aspects of the trajectory can be emphasized to align with mission-specific priorities^37^. The following sections define and explain each cost component.

*Path length* $F_L$ This term measures the total spatial distance traversed. Minimizing path length is a fundamental objective in nearly all UAV path planning formulations, as shorter paths typically reduce both flight time and energy consumption^33,38^. We compute $F_L$ as the sum of Euclidean distances between successive waypoints:

3 $$\begin{aligned} \begin{aligned} F_L&= \sum _{k=1}^{N} \Vert P_k - P_{k-1} \Vert _2 \\&= \sum _{k=1}^{N} \sqrt{(x_k - x_{k-1})^2 + (y_k - y_{k-1})^2 + (z_k - z_{k-1})^2}. \end{aligned} \end{aligned}$$

This formulation encourages shorter trajectories that enhance efficiency.

*Smoothness* $F_S$ This term penalizes abrupt directional changes, quantified by the turning angles at intermediate waypoints. UAVs–particularly fixed-wing or high-speed rotary-wing platforms–favor smooth transitions to minimize control effort, reduce aggressive maneuvers, and improve flight stability^39^. Let $\theta _k$ denote the angle between two consecutive path segments at waypoint $P_k$. The smoothness cost is then defined as:

4 $$\begin{aligned} F_S = \sum _{k=1}^{N-1} \theta _k^2. \end{aligned}$$

Squaring the angles places stronger emphasis on sharper turns, guiding the planner toward gentle, continuous paths.

*Altitude regulation* $F_H$ To comply with safety and regulatory constraints, the UAV must maintain altitude within a permissible range during urban flight^33,34,38^. Let $[z_{\min }, z_{\max }]$ be the allowable altitude band. A penalty is incurred for each waypoint $z_k$ outside this range:

5 $$\begin{aligned} F_H = \lambda _{\text {alt}} \sum _{k=0}^{N} \left[ z_k < z_{\min } \vee z_k > z_{\max } \right] , \end{aligned}$$

where the bracketed term evaluates to 1 if the condition is true and 0 otherwise, and $\lambda _{\text {alt}}$ is a large penalty constant. This soft constraint encourages altitude compliance to avoid low-altitude hazards and high-altitude restrictions.

*Energy consumption* $F_E$ UAVs have limited onboard energy, so minimizing energy consumption is critical^35,40^. Vertical motion–particularly frequent climbing or descending–can be energy-intensive. We model this component by summing the absolute altitude differences between successive waypoints, scaled by a constant factor $c_E$:

6 $$\begin{aligned} F_E = c_E \sum _{k=1}^{N} |z_k - z_{k-1}|, \end{aligned}$$

where $c_E$ is a positive weight. This formulation encourages smoother altitude profiles. While simplified, it aligns with the practical goal of reducing energy expenditure without explicitly modeling aerodynamic drag or speed-based losses^40^.

*Wind exposure* $F_W$ Wind effects often intensify with altitude, especially in urban areas^41^. Higher-altitude flight segments are therefore penalized more heavily to account for increased energy demands and potential instability. We define this component as:

7 $$\begin{aligned} F_W = \gamma \sum _{k=1}^{N} z_k \cdot \Vert P_k - P_{k-1} \Vert _2, \end{aligned}$$

where $\gamma$ is a scaling factor. This cost promotes lower-altitude flight within the permissible band, especially in the presence of strong upper-level winds. It complements the altitude regulation term $F_H$ by refining vertical choices based on environmental conditions.

*Obstacle penalty* $F_O$ Collision avoidance is a primary safety requirement in UAV navigation^42^. Ideally, path segments should not intersect any obstacle in $\mathscr {O}$. Let $n_{\text {obs}}$ represent the number of such intersections. The obstacle penalty is defined as:

8 $$\begin{aligned} F_O = M_{\text {obs}} \cdot n_{\text {obs}}, \end{aligned}$$

where $M_{\text {obs}}$ is a large penalty constant. This formulation discourages paths that penetrate obstacle volumes and promotes solutions that are essentially collision-free.

*No-fly zone penalty* $F_Z$ Certain airspaces–such as those around airports or critical infrastructure–are designated as no-fly zones and must be strictly avoided^34^. To enforce this, we apply a fatal penalty to any trajectory intersecting $\mathscr {N}$:

9 $$\begin{aligned} F_Z = {\left\{ \begin{array}{ll} 0, & \text {if no-fly zones are avoided} \\ M_{\text {nfz}}, & \text {otherwise} \end{array}\right. } \end{aligned}$$

where $M_{\text {nfz}}$ is an extremely large constant. This term acts as a hard constraint, ensuring full compliance with airspace restrictions.

Each term in the cost function $J$ represents a distinct operational requirement essential for safe and efficient navigation of UAV in complex urban environments. The weighted-sum formulation allows the planner to balance multiple, often competing objectives, such as minimizing path length, conserving energy, ensuring altitude compliance, and maintaining airspace safety^37,42^. To prevent any single term from dominating the optimization due to differing units and scales, the weighting coefficients $w=\{w_L,w_S,w_H,w_E,w_W,w_O\}$ in Eq. (2) were chosen after preliminary experiments in which each cost component was first normalized by its maximum observed value over a set of random trajectories. This normalization ensured comparable contributions across components, thereby making the weights primarily responsible for encoding mission priorities rather than scale compensation. Following normalization, the weights were set to $w_L=0.25$, $w_S=0.15$, $w_H=0.15$, $w_E=0.15$, $w_W=0.10$, and $w_O=0.20$, reflecting a balanced trade-off between trajectory efficiency and operational safety, in line with the recent UAV path-planning literature^35–44^.

### Improved roosters algorithm

Although the Rooster Algorithm (RA) is inspired by the mating rituals of roosters and chickens, it omits certain behavioral nuances, most notably the rooster’s ’dancing’ display aimed at winning the chicken’s favor. Instead, RA simply compares the fitness score of a rooster with that of a chicken; if the rooster’s fitness exceeds the chicken’s, it is deemed to have successfully impressed her.

To enhance this interaction, the Improved Roosters Algorithm (IRA) incorporates a more sophisticated “dance” based on the Arithmetic Spiral (AS). In polar coordinates the spiral is defined by;

10 $$\begin{aligned} r = a + b\,\theta , \end{aligned}$$

where $r$ is the radial distance from the origin, $\theta$ is the angular coordinate (in radians), and $a, b \in \mathbb {R}$. The parameter $a$ shifts the spiral outward from its center, while $b$ controls the spacing between successive turns.

A point on this spiral can be mapped into the Cartesian plane via;

11 $$\begin{aligned} x&= (v t + c)\,\cos \bigl (\omega t\bigr ),\end{aligned}$$

12 $$\begin{aligned} y&= (v t + c)\,\sin \bigl (\omega t\bigr ), \end{aligned}$$

where $v$ denotes the instantaneous speed at time $t$, $\omega$ is the angular velocity, and $c$ sets an initial offset.

Within IRA, the rooster executes this AS-based trajectory around the stationary chicken, completing two full revolutions. The values of $a$ and $b$ are automatically chosen to ensure exactly two turns given the current rooster–chicken separation. Likewise, $v$ and $\omega$ are calibrated so that the rooster’s position and orientation follow the desired spiral motion over the two-turn cycle. If the rooster occupies a more favorable fitness position relative to the chicken after the dance, it is considered to have captivated her attention.

IRA is a refined metaheuristic designed to solve complex UAV path planning challenges by enhancing both exploration and exploitation phases of the classical RA. Based on the detailed description provided in above, IRA introduces the following key innovations:

- *Hierarchical Population Structure:* Solutions are divided into roosters ($N_r$) and chickens ($N_c$) based on fitness. This preserves elite solutions for intensified search while maintaining diversity among lower-ranked individuals.
- *Spiral Dancing Operator:* For each rooster–chicken pair, we generate a set of candidate points along a spiral path emanating from the rooster toward the chicken. The spiral is parameterized by the angle increment $\Delta \theta$ and expansion factor r, enabling dynamic balance between local refinement and global search.
- *Improved Mating Operator (i_rstr)*: Each chicken produces *k* offspring by recombining coordinates from randomly selected roosters. Offspring vectors that exceed the search bounds $[\textrm{Lb},\textrm{Ub}]^d$ are corrected via boundary mending.
- *Adaptive Parameter Control:* The spiral expansion factor and mating ratio adapt linearly over iterations from exploratory to exploitative regimes.

The algorithm workflow is as follows:

1. *Initialization:* Generate a population P of size $N= N_c + N_r$ uniformly in $[\textrm{Lb},\textrm{Ub}]^d$, evaluate the fitness f(x).
2. *Ranking:* Sort P by the fitness; assign the best $N_c$ as chickens C, and rest $N_r$ as chickens R.
3. *Spiral Dancing (Exploration):* For each $(r_j,c_j)$:
  Compute spiral points: $S_j = \{\,r_j + r(t)\bigl (\cos (\theta _j + t\Delta \theta ),\,\sin (\theta _j + t\Delta \theta )\bigr )\mid t=1{: }T\}$
  Evaluate f on $S_j$, retain the best dancer $d_j$.
4. *Improved Mating (Exploitation): * Apply i_rstr(R, C, k) to generate offspring set O, evaluate f(o) for each $o\in O$.
5. *Selection: * Form new population by selecting the best N individuals from $P \cup \{d_j\}\ \cup O$.
6. *Termination: * Repeat steps 2–5 until maximum iterations or convergence criterion.

Based on the workflow, pseudocode of the algorithm can be written as:

**Algorithm 1:**
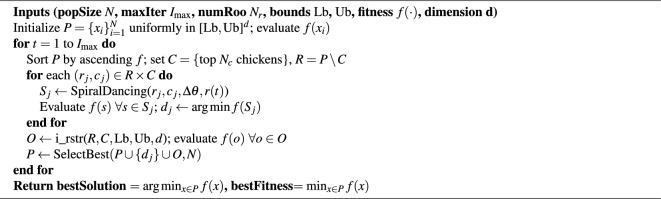
Improved Roosters Algorithm (IRA)

To facilitate a clearer understanding of IRA’s internal mechanics, the algorithm’s three key components are discussed individually below. The spiral exploration mechanism enriches the search diversity by perturbing elite solutions using a logarithmic spiral pattern, enabling the algorithm to escape local minima. The adaptive constraint-handling strategy dynamically adjusts penalty weights according to the current state of constraint violations, ensuring a balanced trade-off between feasibility and optimality throughout the search process. Finally, the hierarchical population structure promotes stable convergence by preserving elite solutions while maintaining exploration through subgroup-based updates. Together, these components enable IRA to achieve both robustness and adaptability in complex constrained environments.

### Computational complexity analysis

From a computational viewpoint, the proposed IRA algorithm follows the standard structure of population-based metaheuristics. Let *N* denote the population size, *T* the maximum number of iterations, and *D* the dimensionality of the solution space. In each iteration, all individuals are updated according to the three core mechanisms of IRA: spiral exploration, adaptive constraint handling, and hierarchical population restructuring. Each of these operators requires a constant or linear number of arithmetic operations with respect to *D*.

Therefore, the total time complexity of IRA can be expressed as:

$$\begin{aligned} \mathscr {O}(N \cdot T \cdot D), \end{aligned}$$

which is consistent with other swarm-based optimizers such as PSO and GWO. Although IRA introduces additional behavioral components compared to the original RA, these mechanisms do not increase the asymptotic order of complexity; they act as constant-factor extensions. In practice, this results in a moderate increase in execution time, which is justified by the significant gains in solution quality and robustness observed in constrained UAV path planning scenarios.

## Experiments and results

In this section, the test setup is described, and the performance of the proposed IRA in obtaining the optimal path is presented in comparison with the SGA, PSO, DE, GWO and the original RA.

A study of metaheuristic algorithms shows that PSO, GA, DE and GWO are among the most cited algorithms^45^; therefore, these algorithms were selected for testing in this paper. To demonstrate the contribution of the article to the literature, RA, the previous version of the proposed algorithm (IRA), was also included in the testing.

### Experimental setup and test conditions

The performance of SGA, PSO, DE, GWO, RA, and the proposed IRA was assessed across three territorial scenarios capturing a spectrum of urban complexity–sparse (small-town), moderate (city-scale), and dense (metropolitan-scale). In each scenario, the UAV must pick up a package at a fixed start point and deliver it to a designated endpoint with minimum cost while avoiding obstacles and no-fly zones. The start point was fixed at $(0,0,0)$. For each scenario, the delivery point was sampled once at random and then held constant across all algorithms to ensure a fair comparison.

**Table 1:** Territorial scenario parameters.

Territories	Search space	Obstacles	No-fly zones
Low density	50x50	20	5
Medium density	100x100	50	10
High density	100x100	70	13

The parameters defining the number of obstacles and no-fly zones for low-, medium-, and high-density scenarios (Table 1) were determined based on typical small town, city, and metropolitan area characteristics reported in previous UAV navigation studies^33,34^. These density levels aim to reflect increasingly complex urban environments in terms of obstacle distribution and airspace restrictions, providing a realistic framework for performance evaluation. The dimensions of the search space were chosen to balance computational feasibility and representativeness of real-world urban sectors, following the guidelines in^35^.

As shown in Table 2, SGA, PSO, DE and GWO parameter settings were selected based on recommendations in^12,46–48^, while RA and IRA parameters were chosen to ensure comparable population structures. Table 3 also displays average elapsed time for all algorithms.

For the experiments, the number of iterations was set to 100. The population size was 50 in the low-density scenario and 100 in the medium-density and high-density scenarios. The implementations of all algorithms were coded in MATLAB 2019a on an 13th Gen Intel Core i7-13700H 2.40 GHz processor. To mitigate stochastic variability, each experiment was repeated 50 times with different random seeds. The minimum cost, median cost, standard deviation, and maximum cost from these runs are reported. The median is preferred over the mean to reduce the influence of outliers.

**Table 2:** The parameter settings of the algorithms.

Algorithm	Parameter	Value
SGA	Tournament size	3
	Crossover rate	0.7
	Mutation probability	0.05
PSO	w	0.2
	c1	2
	c2	2
DE	Crossover rate	0.7
	Scaling factor	0.8
GWO	A	[-1, 1]
	C	[0, 2]
RA	Rooster’s size	4
IRA	Number of roosters	4

**Table 3:** The average elapsed time of the algorithms.

Algorithm	Elapsed time (second)
SGA	4.479774
PSO	2.734634
DE	1.102536
GWO	1.336672
RA	6.429520
IRA	8.164728

Friedman and Wilcoxon Signed Rank tests were also employed to display the significance of the results. IBM SPSS Statistics 22 was performed to acquire the Friedman test results while the MATLAB 2019a *signrank* function was utilized to obtain the Wilcoxon signed rank test results.

### Results

#### Test results

In addition to quantitative metrics, representative visualizations of the best path planning outputs were generated for each algorithm, both in 3D and 2D. All algorithms were tested under the same obstacles and no-fly zones. The rotated perspectives confirm that the generated trajectories successfully avoided collisions with obstacles.

Tables 4, 5, and 6, with the best performance values ​​highlighted in bold, present a detailed summary of the cost statistics obtained for all algorithms under low-, medium-, and high-density scenarios, respectively:

Notably, IRA attains the best central- and best-case performance in the low-density test: it yields the smallest minimum cost (12,099) and the lowest medians (12,293), while displaying the tightest dispersion case (std = 181.02). Both PSO and GWO delivered competitive results in this scenario, particularly in terms of minimum cost, but neither could consistently outperform IRA in terms of median performance or robustness.

For the medium-density case, the original RA showed highly unstable behavior, with frequent collisions and violations leading to infeasible trajectories and extremely large cost values, which significantly inflated both the median and standard deviation. DE also suffered from severe instability, frequently producing infeasible solutions that resulted in disproportionately large cost metrics. Although PSO and GWO occasionally produced competitive solutions, its variability across runs remained high. IRA, on the other hand, maintained its relative advantage, achieving the smallest minimum cost and the lowest median cost (12,976 and 13,547, respectively), and exhibiting moderate dispersion, indicating greater resilience to the increased environmental complexity.

In the high-density setting the landscape becomes substantially more constrained. GWO reports the smallest minimum (14,912), but this single best outcome contrasts sharply with its extremely large median and inflated dispersion (many infeasible/high-cost runs), indicating that the low minimum arises from a few isolated feasible solutions rather than consistent performance. By contrast, IRA maintains the lowest median cost across all densities (20,694 in the high-density case) and delivers competitive dispersion (std = 460,880), despite a single high-cost outlier caused by a collision/no-fly violation. This pattern–consistently small medians combined with moderate-to-competitive standard deviations–indicates that IRA is both effective at finding high-quality solutions and more reliable across repeated trials under increasingly stringent constraints.

Across all density scenarios, IRA consistently achieved the lowest median cost values, indicating stable central tendency advantages compared to standard metaheuristics. Its competitive standard deviations further confirm its robustness and consistent performance across multiple runs. These results collectively highlight the benefits of the improvement mechanisms introduced in IRA, particularly in environments where constraint handling and search stability are critical.

**Table 4:** Summary of cost metrics under low density.

Algorithm	Min cost	Median cost	Std dev	Max cost
SGA	13085	15239	916.97	17502
PSO	12647	14985	993.40	16581
DE	13021	13910	565.97	15545
GWO	12562	13147	335.92	14067
RA	17446	19941	1460.50	23617
IRA	**12099**	**12293**	181.02	12906

**Table 5:** Summary of cost metrics under medium density.

Algorithm	Min cost	Median cost	Std dev	Max cost
SGA	14118	16583	909.70	19238
PSO	14918	15997	3372.40	32535
DE	17133	1012600	496890	1012600
GWO	13808	14042	429730	1012600
RA	29192	1036300	546360	2033300
IRA	**12976**	**13547**	327980	1013400

**Table 6:** Summary of cost metrics under high density.

Algorithm	Min cost	Median cost	Std dev	Max cost
SGA	20893	40820	485000	1017100
PSO	16670	29505	497250	1018400
DE	32725	1038200	612740	2040100
GWO	**14912**	1013300	429760	1013500
RA	15214	22156	488720	1021600
IRA	15393	**20694**	460880	1021800

**Fig. 2:**
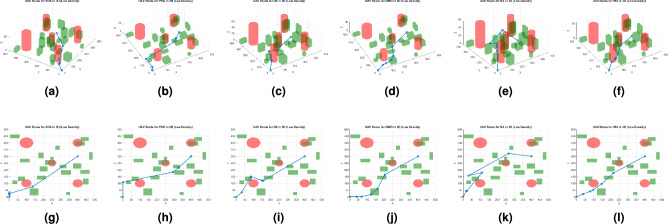
Best path planning outputs under low density. Labels (**a**), (**b**), (**c**), (**d**), (**e**), and (**f**) show the 3D results of the SGA, PSO, DE, GWO, RA, and IRA algorithms, respectively, while labels (**g**), (**h**), (**i**), (**j**), (**k**), and (**l**) show the 2D results of the SGA, PSO, DE, GWO, RA, and IRA algorithms, respectively.

**Fig. 3:**
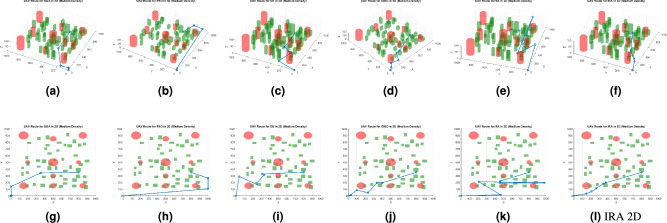
Best path planning outputs under medium density. Labels (**a**), (**b**), (**c**), (**d**), (**e**), and (**f**) show the 3D results of the SGA, PSO, DE, GWO, RA, and IRA algorithms, respectively, while labels (**g**), (**h**), (**i**), (**j**), (**k**), and (**l**) show the 2D results of the SGA, PSO, DE, GWO, RA, and IRA algorithms, respectively.

**Fig. 4:**
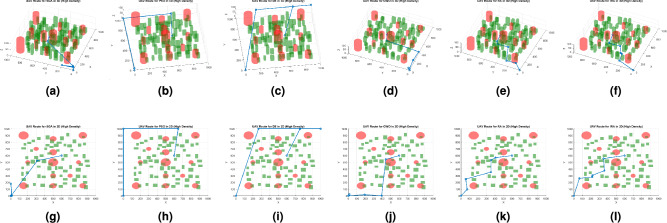
Best path planning outputs under high density. Labels (**a**), (**b**), (**c**), (**d**), (**e**), and (**f**) show the 3D results of the SGA, PSO, DE, GWO, RA, and IRA algorithms, respectively, while labels (**g**), (**h**), (**i**), (**j**), (**k**), and (**l**) show the 2D results of the SGA, PSO, DE, GWO, RA, and IRA algorithms, respectively.

Figures 2, 3, and 4 present the best path planning outcomes under three obstacle density conditions. Each figure combines 3D (top row) and 2D (bottom row) visualizations for SGA, PSO, DE, GWO, RA, and IRA, providing a comprehensive comparison of trajectory behavior across methods.

In the low-density scenario (Fig. 2), all algorithms successfully identify feasible paths; however, IRA and GWO produce smoother and more direct trajectories, minimizing redundant deviations. DE, while competitive in path length, occasionally exhibits sharper transitions due to its aggressive exploration mechanism.

Under medium-density conditions (Fig. 3), IRA continues to demonstrate balanced altitude control and efficient obstacle avoidance. GWO maintains similar consistency, reflecting its strong equilibrium between exploration and exploitation. In contrast, DE tends to oscillate near obstacle boundaries, and SGA occasionally generates locally trapped routes.

In high-density environments (Fig. 4), IRA again outperforms others in maintaining smooth directional changes and avoiding abrupt altitude fluctuations. GWO also performs robustly, yielding compact and adaptive paths with fewer oscillations compared to PSO and DE. Conversely, RA and SGA show higher path irregularity and longer overall distances, indicating reduced adaptability in constrained spaces.

Overall, visual analysis confirms that IRA and GWO deliver the most stable and cost-effective trajectories, effectively balancing obstacle avoidance with global path efficiency. DE demonstrates competitive performance in exploration but lacks smoothness in complex scenarios, while PSO, SGA, and RA show comparatively higher variance and path distortion.

IRA explicitly optimizes path smoothness as a core objective within the trajectory planning process. The multi-objective cost function includes a smoothness term that penalizes abrupt directional changes based on the turning angles between consecutive waypoints. The corresponding weight was carefully tuned to achieve an appropriate trade-off among path smoothness, efficiency, obstacle clearance, and altitude regulation. As demonstrated in Figs.  2, 3 and 4, IRA consistently produces smoother and more direct trajectories than the baseline algorithms, exhibiting minimal redundant deviations and maintaining stable directional transitions across varying obstacle densities. It is important to note that no post-processing or spline-based smoothing was applied to the results, as the observed smoothness arises intrinsically from the optimization process itself.

As observed in Tables 5 and 6, both the standard deviation and maximum cost values increase significantly in the medium- and high-density scenarios. This behavior is not indicative of instability in the optimization process, but rather a consequence of catastrophic failures occurring in a limited number of runs. Specifically, when an algorithm generates a trajectory that violates a no-fly zone or collides with an obstacle, a very large penalty is imposed, resulting in an extreme total cost. Such penalized cases inflate the maximum and dispersion metrics, even if the majority of runs remain feasible.

However, it is important to emphasize that median cost–representing the central tendency and typical performance–remains low for IRA across all density settings, confirming its robustness against constraint-induced failures. In contrast, algorithms such as DE and RA exhibit not only higher variance but also higher median values, indicating that their instability is systemic rather than sporadic.

These observations highlight a critical challenge in UAV path planning under strict urban constraints: even a single infeasible decision can lead to disproportionate penalties. Future work will therefore focus on incorporating feasibility-repair operators and adaptive penalty control to reduce catastrophic failures while preserving exploration capability.

#### Statistical results

Hypothesis testing has been commonly used to infer the comparison of the algorithms^49^. However, the null hypothesis, H_0_, and the alternative hypothesis, H_1_, are supposed to be defined in order to make inferences. The null hypothesis is a proposition that generally refers no differences between compared algorithms while the alternative hypothesis is an expression of the differences:

##### H_0_

There is no difference between compared algorithms.

##### H_1_

There is difference between compared algorithms.

Moreover, the probability value of a statistical test, $\alpha$, decides which hypothesis should be rejected. For our test, $\alpha$ is accepted as 0.05.

As a non-parametric statistical test, The Friedman^50,51^ has been utilized to specify differences in the manner of algorithms behavior. The method initiates its procedure by giving rank to each row based on the values of columns in the row, then, it computes the total rank values for each column. The significance of the test is determined by ${X}^{2}$, called Chi-square, distribution with k-1 degrees of freedom (df) where k is the number of compared algorithms. Appropriate chi-square values corresponding to df can be found in^52^.

From the table in^52^, expected ${X}^{2}$ = 11.07 since df = 5 and $\alpha =0.05$. If found ${X}^{2}$ value is greater than the expected one, then, we need to reject the null hypothesis.

**Table 7:** Mean ranks of the methods for the Friedman.

Algorithm	Low density	Medium density	High density
SGA	4.42	3.08	3.50
PSO	4.06	2.90	3.40
DE	3.40	4.65	5.76
GWO	2.12	2.84	3.06
RA	6.00	5.93	2.86
IRA	1.00	1.60	2.42

**Table 8:** Statistical values for the Friedman test.

Statistical value	Low density	Medium density	High density
Chi-Square	222.491	169.578	98.389
prob_value	4,347E-46	8.9779E-35	1.1551E-19

The Friedman test results are summarized in Tables 7 and 8. The mean ranks reported in Table 7 clearly indicate performance differences among the algorithms. An algorithm with a lower mean rank corresponds to better overall performance across the benchmark runs. Accordingly, IRA achieves the lowest mean ranks in all density scenarios (1.00, 1.60, and 2.42 for low, medium, and high density, respectively), indicating that it consistently outperforms the competing algorithms. In contrast, RA and DE exhibit relatively high mean ranks, reflecting weaker performance, particularly under medium- and high-density conditions.

Table 8 presents the test statistics obtained from the Friedman test. For all density scenarios, the computed chi-square (${X}^{2}$) values (222.49, 169.58, and 98.39, respectively) exceed the critical value of 11.07 at the significance level of $\alpha =0.05$. In addition, all associated probability values (prob_values) are far below 0.05, strongly rejecting the null hypothesis that “There is no difference between compared algorithms.” These findings confirm that the observed performance differences across algorithms are statistically significant in all cases.

However, the Friedman test alone does not specify which pairs of algorithms differ significantly. Therefore, the Wilcoxon signed-rank test is subsequently applied to perform pairwise comparisons and to identify the exact sources of these differences.

The Wilcoxon signed rank test is another non-parametric statistical test that has been used to state the differences between two samples or algorithms^53^. In this paper, it is performed in pairs: one algorithm is systematically selected from the first column of the table and then compared with all subsequent algorithms. In the tables, the p values indicate the degree of similarity between the compared algorithms. “0” implies “no similarity” between the outcomes of the compared algorithms, while “1 indicates that they are identical.

**Table 9:** The results of Wilcoxon signed rank test in low density.

Algorithms	SGA	PSO	DE	GWO	RA	IRA
SGA	1	0.1309	7.5569e-10	7.5569e-10	3.0382e-07	7.5569e-10
PSO		1	7.5569e-10	7.5569e-10	6.6921e-06	1.7569e-09
DE			1	7.5569e-10	7.5569e-10	7.5569e-10
GWO				1	7.5569e-10	7.5569e-10
RA					1	1.1303e-08
IRA						1

**Table 10:** The results of wilcoxon signed rank test in medium density.

Algorithms	SGA	PSO	DE	GWO	RA	IRA
SGA	1	0.2527	7.5569e-10	6.9099e-04	9.0681e-10	0.0431
PSO		1	7.5569e-10	6.6704e-04	4.0126e-09	0.1689
DE			1	1.1548e-09	1.3091e-08	7.5569e-10
GWO				1	6.6267e-08	3.6788e-06
RA					1	6.5574e-06
IRA						1

**Table 11:** The results of wilcoxon signed rank test in high density.

Algorithms	SGA	PSO	DE	GWO	RA	IRA
SGA	1	0.2220	2.6611e-09	0.1573	0.0144	0.0010
PSO		1	1.7742e-08	0.3516	0.2527	0.0126
DE			1	4.7902e-08	1.6552e-09	2.6611e-09
GWO				1	0.0814	0.0068
RA					1	0.1167
IRA						1

Tables 9, 10 and 11 demonstrate the performance of all algorithms in the Wilcoxon Signed Rank Test. This statistical evidence strongly supports the existence of significant performance differences among the tested algorithms. Particularly, the proposed IRA algorithm consistently exhibits statistically superior results compared to the classical RA and the other algorithms across all density levels, as indicated by its very low *p-values* (often below 0.01). This outcome confirms that the improvements introduced in the IRA–such as its adaptive update mechanism and enhanced exploration–exploitation balance–contribute to its robustness and optimization capability. Furthermore, since most pairwise comparisons yield *p-values* very close to 0, the null hypothesis of equal median performance can be confidently rejected. Hence, it can be concluded that the proposed IRA algorithm achieves a statistically significant enhancement over the existing algorithms under varying problem densities.

## Conclusion

Determining optimal and feasible routes is a central challenge for the autonomous deployment of UAVs in dense urban environments, where navigation must balance path efficiency, energy consumption, and strict regulatory constraints. This paper proposes the Improved Roosters Algorithm (IRA), a novel extension of the original RA that introduces new mechanisms for exploration, constraint-handling, and convergence control.

Comprehensive experiments were conducted across a spectrum of urban complexity–from sparse to highly cluttered–under realistic operational constraints with obstacles and no-fly zones. The results show that IRA consistently achieves lower median cost than baseline algorithms such as SGA, PSO, DE, GWO and RA. Notably, in the most cluttered setting, IRA outperforms its closest competitor, underscoring its robustness under the most challenging conditions. Beyond numerical gains, qualitative analyses indicate that IRA generates smoother, more regulation-compliant trajectories, avoiding excessive detours, sharp turns, and infeasible paths that frequently occur in competing methods. These findings highlight IRA’s ability to maintain both feasibility and efficiency in constraint-rich environments, which is critical for practical UAV operations.

While this study provides strong simulation-based validation, its implications extend to real-world UAV missions. Applications such as drone-based logistics, emergency response, and infrastructure inspection can benefit from IRA’s capacity to generate safe, efficient, and regulation-compliant paths in complex cityscapes. By embedding operational constraints directly into the optimization process, IRA moves beyond incremental algorithmic improvements toward solutions that are more readily translatable to real-world deployment.

## Limitations and future work

While the proposed IRA framework demonstrates promising performance, several limitations should be acknowledged. First, IRA incurs a relatively higher computational cost compared to baseline algorithms due to the additional operators introduced. Although this overhead is acceptable for offline planning, future work will focus on parallelization and algorithmic simplifications to enable real-time applications. Second, the current implementation is in MATLAB, which is suitable for prototyping but not optimized for speed; future implementations in compiled languages such as C++ or Python are expected to improve scalability.

A further limitation arises from the cost variability observed in medium- and high-density environments, where certain runs resulted in extreme penalty values due to collisions or no-fly zone violations. These catastrophic failures inflate standard deviation and maximum cost metrics, highlighting the need for feasibility-repair operators or adaptive penalty refinement to reduce the impact of infeasible trajectories.

Additionally, the experimental scenarios were limited to synthetic urban environments, which may not fully reflect real-world complexities; incorporating benchmark and realistic maps is a priority for future validation. Finally, the sensitivity analysis of the weight parameters was limited in scope; more extensive multi-factorial analyses will be conducted to deepen the understanding of parameter interactions and algorithm robustness.

## Data Availability

The datasets used and/or analysed during the current study available from the corresponding author on reasonable request.
